# Commentary: Bullous pemphigoid associated with COVID-19 vaccines: An Italian multicenter study

**DOI:** 10.3389/fmed.2022.1096867

**Published:** 2022-12-19

**Authors:** Michael Kasperkiewicz, Stefan Tukaj

**Affiliations:** ^1^Department of Dermatology, Keck School of Medicine, University of Southern California, Los Angeles, CA, United States; ^2^Department of Molecular Biology, Faculty of Biology, University of Gdańsk, Gdańsk, Poland

**Keywords:** autoimmune bullous diseases, bullous pemphigoid, COVID-19, SARS-CoV-2, vaccination

## Introduction

Autoimmune bullous diseases (AIBDs), including their main subtype bullous pemphigoid (BP), represent autoantibody-mediated, chronic inflammatory mucocutaneous blistering disorders which partly require potent and long-term immunosuppressive therapies (1, 2). Patients with AIBDs have faced considerable challenges and had to be closely monitored by specialized dermatologists during the COVID-19 pandemic (3–5).

The major advance in combating COVID-19 has been the advent of SARS-CoV-2 vaccines including the next-generation mRNA-based platforms by Pfizer-BioNTech and Moderna. According to international expert recommendations for the management of AIBDs in the COVID-19 era, it has been advised that every patient without contraindications to vaccination is immunized with one of the authorized vaccines to prevent from SARS-CoV-2 infection (5). However, with global mass vaccinations being performed, there have been several reports, including an Italian multicenter study by Maronese et al. (6), on the possible but so far unproven induction of new-onset or exacerbation of pre-existing AIBDs by COVID-19 vaccines (7).

## Subsections relevant for the subject

Maronese et al. (6) reported on the collection of clinical, histopathological, and immunopathological data of 21 patients with COVID-19 vaccine-associated BP (9 females and 12 males, median age: 82 years). Seventeen patients received the Pfizer-BioNTech vaccine, two the Moderna vaccine, one the AstraZeneca adenoviral vector vaccine, and one received the first dose with the AstraZeneca vaccine and the second dose with the Pfizer-BioNTech vaccine. The median latency time between the first vaccine dose and the onset of cutaneous manifestations was 27 days, and the median BPDAI at onset was 42. Eleven out of 17 patients (65%) had positive anti-BP180 autoantibodies (median value: 106.3 U/mL), whereas only five out of 17 (29%) were positive for anti-BP230 autoantibodies (median value: 35.3 U/mL). The authors concluded that in terms of mean age, disease severity at diagnosis, and clinical phenotype, vaccine-associated BP patients are similar to idiopathic BP with an overall benign course with appropriate treatment, although the slight male predominance and the reduced humoral response to BP230 may represent peculiar findings.

## Discussion

As of to date, the presumed association between COVID-19 vaccination and AIBDs seems to be a rare event or even a random coincidence (8). According to a systematic review, out of more than 900 published post-SARS-CoV-2-vaccinal cases (mostly mRNA vaccines), only about 6% presented clinically with new AIBDs and 10% had a flare or worsening of pre-existing AIBDs being usually well-controlled with standard immunosuppressive treatment, whereas vaccination did not negatively influence the clinical course in all remaining patients (7). Of note, data on the relationship between COVID-19 vaccination and AIBDs are mostly derived from single case reports with a low level of evidence and a cross-sectional study limited by subjective patient self-reports (7). In addition, an epidemiological study involving over 1.5 million people with mRNA COVID-19 vaccination found no difference in risk of new-onset BP among persons receiving the mRNA COVID-19 vaccine within 6 months compared to non-vaccinated, matched control cohorts, although a misclassification bias from the use of ICD codes of the database source for the diagnosis of BP has been a limitation (9). The conclusion of the study by Maronese et al. (6) that COVID-19 vaccine-associated BP patients basically do not differ from idiopathic BP matches the aforementioned findings. Although the minimal male predominance and the reduced autoantibody response to BP230 were considered unique features of post-SARS-CoV-2-vaccinal BP cases, this needs to be interpreted with caution due to the low number of investigated patients.

The reported time between receiving the first or second dose of the COVID-19 vaccine and manifestation of AIBDs ranges between 1 day and 6 weeks (7). In the study by Maronese et al. (6), the median latency time was 6.5 (IQR: 4–7) days after the first dose and 7 (IQR: 4–14.5) days from the second dose. As discussed in that study, the latency time shorter than a week since the first dose (i.e., the minimum time required for antibody production) argues against a *de novo* antibody response-driven pathology and raises the question about other putative mechanisms by which COVID-19-vaccines may potentially induce AIBDs.

In this context, it is worth mentioning that we have recently shown that circulating anti-SARS-CoV-2 antibodies (generated either by SARS-CoV-2 infection or SARS-CoV-2 vaccination) do not cross-react with AIBD autoantigens including BP180/230, discrediting the previously suggested molecular mimicry theory at least at the humoral level (6, 10). The possibility of molecular mimicry activating cross-reactive T cells cannot be excluded. It can be also hypothesized that SARS-CoV-2 immunization through vaccination or infection may induce or aggravate autoimmunity in genetically predisposed persons by alternative modalities such as bystander activation of immune cells (e.g., stimulation of pre-existent, sub-clinical autoreactivity). In fact, several molecular mechanisms linking coronavirus exposure and other immune-mediated diseases (e.g., multiple sclerosis, rheumatoid arthritis, systemic lupus erythematosus, idiopathic thrombocytopenic purpura, and inflammatory bowel disease) have been proposed (11, 12). Of note, there are also single reports of BP, including infantile BP, following not only SARS-CoV-2 vaccination but also COVID-19 (13, 14).

## Conclusion

In conclusion, further knowledge regarding COVID-19 vaccination and AIBDs has been gained since the publication of the study by Maronese et al. (6). More recent evidence-based epidemiological and mechanistic data from larger cohorts argue against a direct link between COVID-19 vaccination and AIBDs, at least in the vast majority of cases. Nevertheless, individual subjects prone to develop AIBDs in association with SARS-CoV-2 vaccines may exist. While additional well-designed investigations on this controversial topic are required, SARS-CoV-2 vaccination in AIBD patients should be generally encouraged.

## Author contributions

MK drafted the manuscript. ST edited the manuscript. Both authors contributed to the article and approved the submitted version.
